# Modulation of High-Fat Diet-Induced Brain Oxidative Stress by Ferulate-Rich Germinated Brown Rice Ethyl Acetate Extract

**DOI:** 10.3390/molecules27154907

**Published:** 2022-07-31

**Authors:** Nur Hanisah Azmi, Norsharina Ismail, Mustapha Umar Imam, Der Jiun Ooi, Siti Nur Hazwani Oslan

**Affiliations:** 1 Faculty of Food Science and Nutrition, Universiti Malaysia Sabah, Kota Kinabalu 88400, Sabah, Malaysia; snhazwanioslan@ums.edu.my; 2 Natural Medicines and Products Research Laboratory, Institute of Bioscience, Universiti Putra Malaysia (UPM), Serdang 43400, Selangor, Malaysia; 3 Centre for Advanced Medical Research and Training, Usmanu Danfodiyo University, Sokoto P.M.B. 2346, Nigeria; mustapha.imam@udusok.edu.ng; 4 Department of Oral Biology and Biomedical Sciences, Faculty of Dentistry, MAHSA University, Jenjarom 42610, Selangor, Malaysia; djooi@mahsa.edu.my

**Keywords:** germinated brown rice, brain oxidative stress, inflammation, acetylcholinesterase, Alzheimer’s disease

## Abstract

The oxidative stress resulting from the production of reactive oxygen species plays a vital role in inflammatory processes and is associated with neurodegenerative changes. In view of the ability of germinated brown rice (GBR) to improve learning and memory, this present study aimed to investigate the mechanistic basis of GBR’s neuroprotection in a high-fat diet (HFD)-induced oxidative changes in adult Sprague–Dawley rats. Ferulate-rich GBR ethyl acetate extract (GBR-EA; 100 mg/kg and 200 mg/kg body weight) was supplemented orally for the last 3 months of 6 months HFD feeding during the study. GBR-EA supplementation was found to improve lipid profile and serum antioxidant status, when compared to the HFD group. Elevated mRNA expressions of SOD1, SOD2, SOD3, Catalase, and GPX were demonstrated in the frontal cortex and hippocampus of GBR-EA treated animals. The pro-inflammatory changes induced by HFD in the hippocampus were attenuated by GBR-EA through the downregulation of CRP and TNF- α and upregulation of PPAR-γ. GBR also reduced the hippocampal mRNA expression and enzyme level of acetylcholinesterase. In conclusion, this study proposed the possible transcriptomic regulation of antioxidant and inflammation in neurodegenerative processes resulting from high cholesterol consumption, with an emphasis on GBR’s potential to ameliorate such changes.

## 1. Introduction

Reactive oxygen species (ROS) are continuously produced in the body via mitochondrial bioenergetics and oxidative metabolism, and they play a key role in the development of many diseases [1,2]. Increased dietary fat consumption contributes to obesity, which results in a chronic state of inflammation via the formation of white adipose tissue that secretes proinflammatory factors [3]. Furthermore, hypercholesterolemia is a metabolic disorder characterized by elevated total cholesterol levels in the blood, which can be caused by an unbalanced diet, obesity, inherited (genetic) diseases, or other diseases [4]. According to large clinical studies, hypercholesterolemia affects a significant population of adults in developed countries, as evidenced by this prevalence estimate. With a global population of 7.7 billion people in 2019, approximately 25 million people may have familial hypercholesterolemia [5].

Hypercholesterolemia is firmly linked to a pattern of chronic inflammation and has now been shown to have adverse effects on brain physiology and function, which is linked to neurodegenerative illnesses such as Alzheimer’s disease (AD) [6]. In afflicted regions of the brain from AD patients, aberrant pro-inflammatory cytokine production, activation of the inflammatory signaling cascade, acute-phase proteins, and other mediators have been identified [7,8]. Epidemiological research found that diets high in saturated fats were connected with an increased risk of developing endothelial dysfunction and AD [9,10]. Furthermore, patients with high cholesterol are found to be more likely to develop cognitive impairment, which is defined by a gradual deterioration in memory and other cognitive and executive capabilities [11]. Another study revealed that if the total cholesterol in the brain membrane rises, synapses do not function normally, affecting cognitive degradation in AD [12]. Moreover, an elevated low-density lipoprotein cholesterol (LDL-C) level was an independently associated risk factor for the development of AD. The pooled effect size revealed a substantial increase in the risk of AD for people with higher LDL-C levels [13]. According to previous research, elevated levels of low-density lipoprotein cholesterol (LDL-C) and total cholesterol (TC) cause the extracellular deposition of amyloid-β protein (Aβ), obstructing neuronal synaptic connections in the brain and increasing the risk of AD, as well as being associated with worse cognitive function [13,14]. Additionally, high cholesterol has been shown to affect the cholinergic system by decreasing ACh and choline acetyltransferase activity, while boosting AChE activity in the brain [15,16]. The usage of cholesterol-lowering medicines may have slowed the progression of AD [11], implying that they may provide protection against dementia [17].

Hypercholesterolemia is largely influenced by nutrition, hence many approaches to managing the condition center on changing one’s diet. It has been proven that food intake adjustment does not have to be substantial to have favorable long-term effects [11,18], and such changes are rather easy to implement. Dietary adjustments, such as increasing DHA intake or consuming foods rich in flavonoid, can help lower cholesterol and reduce the incidence of AD [18,19]. Oral DHA supplementation in animal tests has been shown to lessen the progression of Alzheimer’s-like brain degeneration [20]. Germinated brown rice (GBR) has numerous bioactives that contribute to its antioxidant effects [21]. Many of GBR’s functional properties can be attributed to its high antioxidant content, which includes ferulic acid, oryzanol, and gamma-aminobutyric acid (GABA), and which is increased during the germination process [22]. Antioxidant-rich foods have shown promise for the prevention of neurodegenerative illnesses such as AD [23]. GBR has also been shown to increase brain function, notably memory and learning [22,24]. Despite this, there is no evidence of the process at work. Thus, this study aimed to evaluate the effects of GBR extract on indicators of oxidative stress and inflammation in the brain of an in vivo hypercholesterolemia model of sporadic AD, as well as the mechanism of action.

## 2. Materials and Methods

### 2.1. Reagents

Brown rice of Malaysian mixed varieties was procured from PadiBeras Nasional (BERNAS) factory (Sri Tiram Jaya, Selangor, Malaysia). Hydrogen peroxide (H_2_O_2_) was purchased from Bendosen Laboratory Chemicals (Selangor, Malaysia) and sodium hypochlorite (NaOCl) was from Dexchem Industries Sdn. Bhd. (Penang, Malaysia. An AChE ELISA (enzyme-linked immunosorbent assay) kit was purchased from Elabscience Biotechnology Co., Ltd. (Wuhan, Hubei, China). A Total RNA Extraction kit was purchased from RBC Bioscience Corp. (Taipei, Taiwan), and a GenomeLab™ GeXP Start Kit was from Beckman Coulter Inc. (Miami, FL, USA). MgCl2 and deoxyribonucleic acid (DNA) Taq polymerase were purchased from Thermo Fisher Scientific (Pittsburgh, PA, USA), Simvastatin was purchased from Pfizer (New York, NY, USA), and Donepezil hydrochloride was purchased from Cayman Chemical (Ann Arbor, MI, USA). Cholesterol and Bradford reagent were purchased from Amresco (Solon, OH, USA). Cholic acid was purchased from Santa Cruz Biotechnology (Santa Cruz, CA, USA). Analytical grade ethanol was purchased from Merck (Darmstadt, Hesse, Germany). Palm oil and standard rat pellets were from Yee Lee Edible oils Sdn. Bhd. (Ipoh, Perak, Malaysia) and Specialty Feeds (Glen Forrest, WA, Australia), respectively. RCL2 Solution was purchased from Alphelys (Toulouse, Plaisir, France). Lipid profile kits were from Randox Laboratories Ltd. (Crumlin, County Antrim, UK).

### 2.2. Germination of Brown Rice and Extraction

Germination of brown rice was carried out according to a previous publication. The ground powder of germinated brown rice was subjected to ethyl acetate (1:4 *w*/*v*) extraction. The germinated brown rice ethyl acetate extract (GBR-EA) obtained comprised a considerable amount of γ-oryzanols (24-methylene cycloartanyl ferulate, campesteryl ferulate, cycloartenyl ferulate, as well as mixtures of β-sitosteryl ferulate and cycloartanyl ferulate), 2MHQ, cinnamic acid, rosmarinic acid, and guaiacol [25,26].

### 2.3. Diet Preparation

A high-fat diet (HFD) with 5% cholesterol was prepared by mixing a certain amount of normal rat pellet, oil, corn start, cholesterol, and cholic acid (Table 1), according to previous studies [27]. A sufficient amount of water was added to the mixture, to bind the components together. The HFD was cut into smaller pieces and dried in an incubator at 50 °C for 24 h, and fed to the rats according to their respective groups.

### 2.4. Animal Experiments

Seventy (70) 2-month-old, male Sprague–Dawley rats weighing between 250 and 280 g were used in this study. Rats were individually housed in stainless steel cages in a well-ventilated room with a 12/12-h light/dark cycle at an ambient temperature of 25–30 °C. Experiments were carried out according to the guidelines for the use of animals and approved by the Animal Care and Use Committee of the Faculty of Veterinary Medicines, Universiti Putra Malaysia (Project approval number: UPM/IACUC/AUP-RO64/2013). The rats were divided into 7 groups, with 10 rats per group (n = 10).

After acclimatization, 1 mL of blood was withdrawn through retro orbital as baseline biochemical data. Next, all rats were fed with HFD for 3 months, except for the normal control group. After the induction period, the groups were subjected to respective interventions via oral gavage for another 3 months. Dosages of Probucol, Simvastatin, and Donepezil were chosen based on previous findings [28,29,30].

Simvastatin, Donepezil and GBR-EA extracts, at varying doses required for interventions (Table 1), were prepared in the form of a per ml water suspension/dissolved solution. The actual volume of water suspension/dissolved solution administered was subsequently adjusted based on the animal body weights in kg. The volumes used in the present interventions were in the range of 1.1–2.0 mL. As for Probucol, owing to its poor wettability, the drug was first dispersed in distilled water via sonication at a concentration of 25 mg/mL. Subsequently, 8 mL/kg of the prepared Probucol (in the range of 2.40–3.80 mL based on the animal body weight) was administered via the oral route [28].

Intervention lasted for 24 weeks in total. Body weights were measured weekly, while food intake was measured daily. At the end of the experiment, the animals were fasted overnight and sacrificed by decapitation under pentobarbital intraperitoneal anaesthesia (0.5 mg/g body weight). The blood was collected in serum separator tubes (BD Vacutainer, Plymouth, UK) via cardiac puncture. The brains, livers, kidneys, and hearts were immediately exteriorized, snap frozen in liquid nitrogen, and kept at −80 °C until analysis.

### 2.5. Determination of Serum Biochemical Profile

Serum total cholesterol, LDL, HDL, triglycerides, and glucose level were measured for all animals following blood collection using Randox analytical kits, according to the manufacturer’s instructions and using a Selectra XL chemistry analyzer instrument (Vita Scientific, Dieren, The Netherlands).

### 2.6. Determination of Serum Total Antioxidant Status

Serum total antioxidant status was determined by ABTS assay. An ABTS radical cation was generated by persulfate oxidation of ABTS. Serum antioxidant was measured by proper mixing 10 μL of a serum sample, 40 μL of ddH_2_O, and 950 μL of ABTS reagent. The mixtures were incubated in the dark at room temperature for 30 min. The absorbance was then read at 734 nm using a Synergy H1 Hybrid Multi-Mode Microplate Reader (BioTek, Winooski, VT, USA). The radical scavenging activity of the serum was measured by the decrease in the absorbance, and % of radical scavenging activity was calculated using a standard curve (y = 0.736x − 0.132, R2 = 0.9978).

### 2.7. Analysis of Antioxidant and Inflammation-Related Gene Expressions

#### 2.7.1. RNA Extraction

RNA was extracted from frontal cortex and hippocampal tissue using a Total RNA Isolation kit (RBC Bioscience Corp., Taiwan, China) as per the manufacturer’s instructions. The RNA concentration was determined using a NanoDrop spectrophotometer (Thermo Scientific Nanodrop, NanoDrop Technologies, Wilmington, DE, USA). The ratios of A260/230 and A260/280 were used to indicate the purity of the extracted total RNA.

#### 2.7.2. Primer Design

Primers were designed on the GenomeLabeXpress Profiler software, using the Rattus norvegicus sequence adopted from the National Center for Biotechnology Information GenBank Database (http://www.ncbi.nlm.nih.gov/nucleotide/, accessed on 12 November 2013). Genes of interest, housekeeping genes, and the internal control are shown in Table 2. Specificity validation of the nucleotide sequences was performed using NCBI-nucleotide-BLAST. An additional 37 base pairs of universal tag sequences were attached to each forward and reverse primer. The primers were supplied by First Base Ltd. (Selangor, Malaysia), and diluted in 1 X TE Buffer to a concentration of 500 nM for reverse primers and 200 nM for forward primers.

### 2.8. Reverse Transcription and Polymerase Chain Reaction

Reverse transcription (RT) and multiplex PCR of RNA samples (50 ng/μL) were carried out in an XP Thermal Cycler (BIOER Technology, Hangzhou, China), according to a previous publication [25].

### 2.9. GEXP Data Analysis

Data analysis was performed using eXpress Profiler software, as in a previous publication [25]. Normalization was performed with GAPDH, according to the manufacturer’s instructions.

### 2.10. Quantification of Acetylcholinesterase (AChE) Level

Brain AChE level was quantified in the hippocampal brain regions by ELISA using an Elabscience kit as per the manufacturer’s protocol. Briefly, the brain tissue (30 mg) was homogenized thoroughly in 180 μL of phosphate-buffered saline (PBS) and centrifuged at 100× *g* for 15 min at 4 °C. Prior to ELISA analysis, the protein concentrations of all samples were determined by standard Bradford assay [31]. AChE level was quantified using calorimetric sandwich ELISA kits and calculated based on the standard curve obtained.

### 2.11. Statistical Analysis

Data were analyzed using IBM SPSS Statistics (SPSS Inc., Chicago, IL, USA). The results were expressed as mean ± SD. One-way ANOVA and Tukey’s post hoc test were performed to determine the level of significance difference, where *p* < 0.05 was considered significant.

## 3. Result and Discussion

### 3.1. Effects of GBR-EA on Caloric Intake and Body Weight

Table 3 displays the food intake and weight of animals for each group. Food intake was not significantly different among all groups (*p* > 0.05), despite the different dietary interventions. Body weights at the beginning of the dietary feeding regimen were not significantly different among the groups (*p* > 0.05). However, at the end of intervention, increased body weight gain was observed in the HFD group compared with the normal control and other treatment groups. Treatment with Donepezil, Simvastatin, Probucol, and GBR-EA100 and GBR-EA200 showed a trend of lowered body weight changes, even though they were not significantly different compared to HFD group.

The fact that sporadic AD accounts for the majority (>90%) of AD cases, the choice of valid models is important to enable the evaluation of early pathological processes that are often not accessible in patients and that subsequently provide feasibility and aid in target discovery and drug development [32]. Oral cholesterol intake was selected on the basis of previous experiments by other researchers [26,33,34]. One confounding factor in studies utilizing high-fat and/or high-cholesterol diet is that such diets often results in substantial weight gain, which may stimulate Aβ accumulation in the brain region per se [34,35] and may have detrimental effects on cognition [34,36]. GBR’s effects in reducing weight gain were previously documented [37,38], and its anti-obesity effects were found to be through the regulation of pancreatic lipase, decrease in fat accumulation by inhibition of adipocyte differentiation and adipocytokine production, as well as the stimulation of lipolysis on adipocytes [39,40]. The reduction in weight gain for rats treated with GBR-EA in this study could be explained by its moderate effects on pancreatic lipase inhibition, reduced lipid accumulation in adipocytes, and stimulation of lipolysis [39,40].

### 3.2. Effects of GBR and GBR-EA on Serum Biochemical Profile

**Figure 1:**
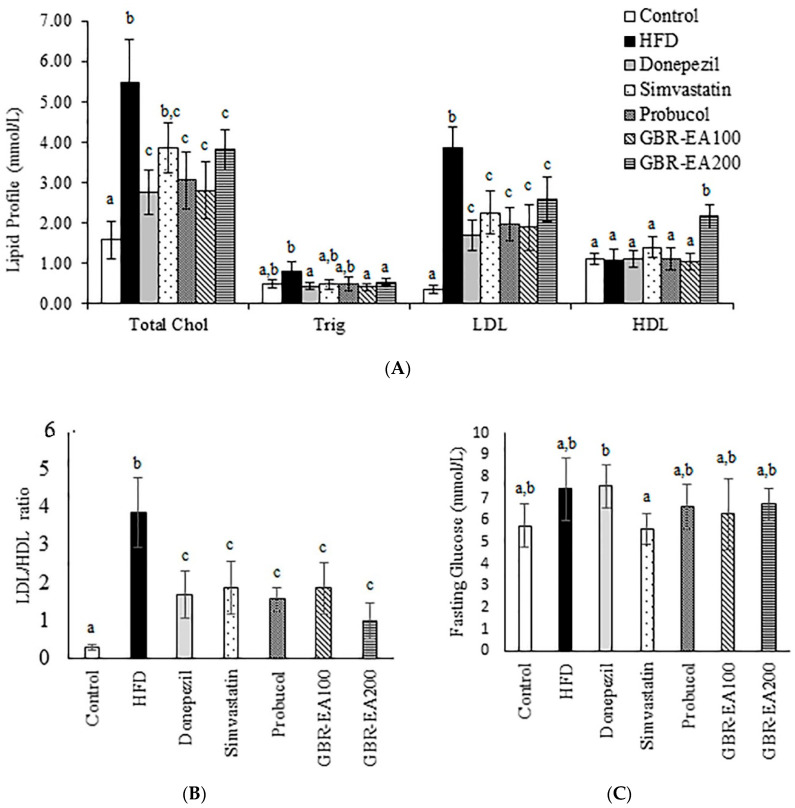
Serum biochemical profile in rats fed with a high-fat diet (HFD) for 6 months, as determined using a chemistry analyzer. (**A**) Lipid profile, (**B**) LDL/HDL ratio, and (**C**) fasting glucose levels were measured in the serum. Values represent the mean ± SD. a–c Mean values with different letters were significantly different among the groups (*p* < 0.05).

Figure 1 shows the biochemical analysis of the serum at the end of the experiment. Total serum cholesterol (TC) differed among the groups, with the HFD group showing the highest elevation in comparison with the normal control groups (*p* < 0.05). Treatment with Probucol, a non-statin anti-hypercholesterolemic drug, showed a better reduction of cholesterol level in comparison with Simvastatin, but this was not statistically significant (*p* > 0.05). Interestingly, the Donepezil group showed a significantly reduced level of TC compared to HFD (*p* < 0.05). The reason behind the reduced serum cholesterol with Donepezil in this study is currently unknown. However, Donepezil was shown to reduce cholesterol accumulation in the brain cells both in vitro and in vivo via the inhibition of cholesterol synthesis [39], this may provide a clue regarding the modulation of cholesterol homeostasis by the drug in this study. LXRs act as a dominant regulator in cholesterol metabolism, including cholesterol synthesis, uptake, and trafficking, and ACh was shown to modulate cholesterol level through activating the LXR pathway [41]. Thus, the modulation of cholesterol homeostasis by Donepezil may be explained through its AChE inhibitory actions.

The treatment with GBR-EA at 100 mg/kg BW and 200 mg/kg BW improved the condition when compared to HFD (*p* < 0.05). Meanwhile, the level of Triglycerides was shown to be elevated for the HFD group in comparison the with normal control group, with no statistically significant differences (*p* > 0.05). However, a significant reduction was shown for Donepezil, GBR-EA100, and GBR-EA200 when compared to the HFD group (*p* < 0.05). Other interventions showed no difference when compared to the HFD group. The LDL level in HFD group was found to be significantly higher when compared to the normal control group (*p* < 0.05). Treatment with Donepezil, Simvastatin, and Probucol showed significant reductions (*p* < 0.05). Similar effects were observed for GBR-EA at 100 mg/kg BW and 200 mg/kg BW.

It appears that the modulation of dietary lipids presents a potential treatment approach that is especially well suited for long-term chronic disease prevention. Unlike conventional pharmaceutical therapies, dietary interventions are essentially devoid of, or at least present fewer, unwanted side effects. The current findings are in agreement with previous reports in which treatment with GBR and its GABA- and ASG-rich extracts lowered the blood cholesterol level in HFD and STZ-induced diabetic rats [38,42]. Several other studies using GBR also reported similar results [43,44]. Previous findings indicated that the bioactives responsible for GBR’s hypocholesterolemic effects include GABA, phytosterol glycosides such as ASG, oryzanol, and phenolics compounds, which were found to be higher in GBR than in brown rice and white rice [42,44,45]. Therefore, it is likely that the hypocholesterolemic effects observed in the current study could be attributed to the presence of compounds such as guaiacol, 2MHQ, rosmaric acid, and γ-oryzanol components, as determined by a previous HPLC analysis [24]. The hypocholesterolemic effects of GBR were suggested to result from the cumulative effects of these reported compounds on the regulation of cholesterol metabolism markers, including ApoA1, LDL-R, lipoprotein lipase, and PPAR-γ [42,44,45].

On the other hand, the level of HDL among all groups was observed to have no significant difference, except for GBR-EA at 200 mg/kg BW, which showed a significant increment (*p* < 0.05). Fasting blood glucose level was not significantly different among the control and HFD groups (*p* > 0.05), even though the HFD group showed a slight elevation. Intervention with Simvastatin showed a reduced glucose level in comparison to HFD, even though this was not significantly different. Probucol, GBR-EA100, and GBR-EA200 did not show significant differences when compared to the control and HFD. Elevated fasting glucose levels and hyperlipidemia were attributed to neurological deficits [33,34,46], thus suggesting the role of glucose intolerance and/or insulin resistance with associated brain inflammation [46]. Even though it is difficult to separate the direct effect of impaired insulin homeostasis on the brain from the accompanying disruption in peripheral and central glucose homeostasis, it is clear that diet-induced alterations in lipid and glucose metabolism, as well as excessive caloric intake, activates certain mechanisms that are detrimental for neuronal plasticity and function.

### 3.3. Effects of GBR-EA on Serum Total Antioxidant Status

**Figure 2:**
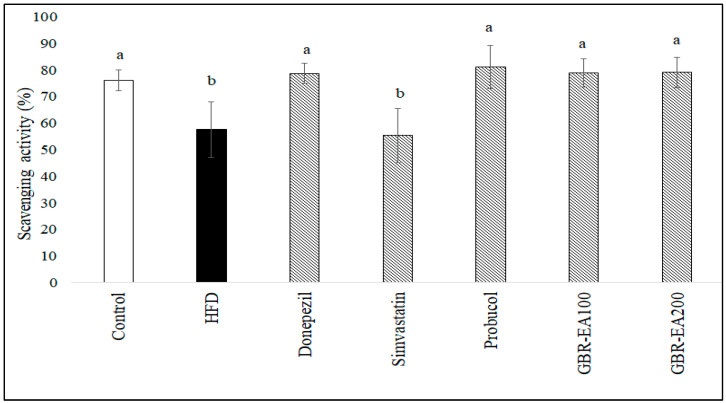
Serum total antioxidant status in rats fed a high-fat diet (HFD) for 6 months, as determined by ABTS assay. Values represent the mean ± SD. a–b Mean values with different letters were significantly different among the groups (*p* < 0.05).

As shown in Figure 2, it was found that the level of ABTS radical scavenging activity in the serum was significantly reduced in HFD-fed rats when compared to the normal control group (*p* < 0.05). This finding revealed that the treatments with Donepezil, Probucol, and 100 mg/kg and 200 mg/kg of GBR-EA enhanced the scavenging activity significantly more than the HFD group (*p* < 0.05). Diet-induced oxidative stress secondary to high glycemic load and/or hypercholesterolemia may have a role in lowering antioxidant status in chronic diseases [47,48]. Chronic sustained hyperglycaemia was previously documented to cause low antioxidant status in a STZ-induced type 2 diabetes rat model fed a high-fat diet, and supplementation with GBR in their diet was found to increase the antioxidant level in the serum, in the same model [44]. The same study revealed that supplementation with white rice did not improve the antioxidant status. Even though a different model was used, the present study provided consistent findings, in which GBR-EA extract was able to improve serum antioxidant status. Maintenance of antioxidant status by GBR suggests that GBR and its extract (100 and 200 mg/kg BW) were able to replenish the supply of antioxidants that maintain serum antioxidant status and/or prevent the deterioration resulting from hypercholesterolemia. GBR’s effects on the improvement of biochemical profile and serum antioxidant status were consistent with the previous report on its antihypercholesterolemic effects in a diabetic rat model [44]. The effect of GBR on antioxidant status herein may have been a result of the higher antioxidant potentials of GBR, and likely due to the higher content of bioactive compounds in GBR [38,44].

### 3.4. Effects of GBR-EA on Antioxidant and Inflammatory Gene Expressions

**Figure 3:**
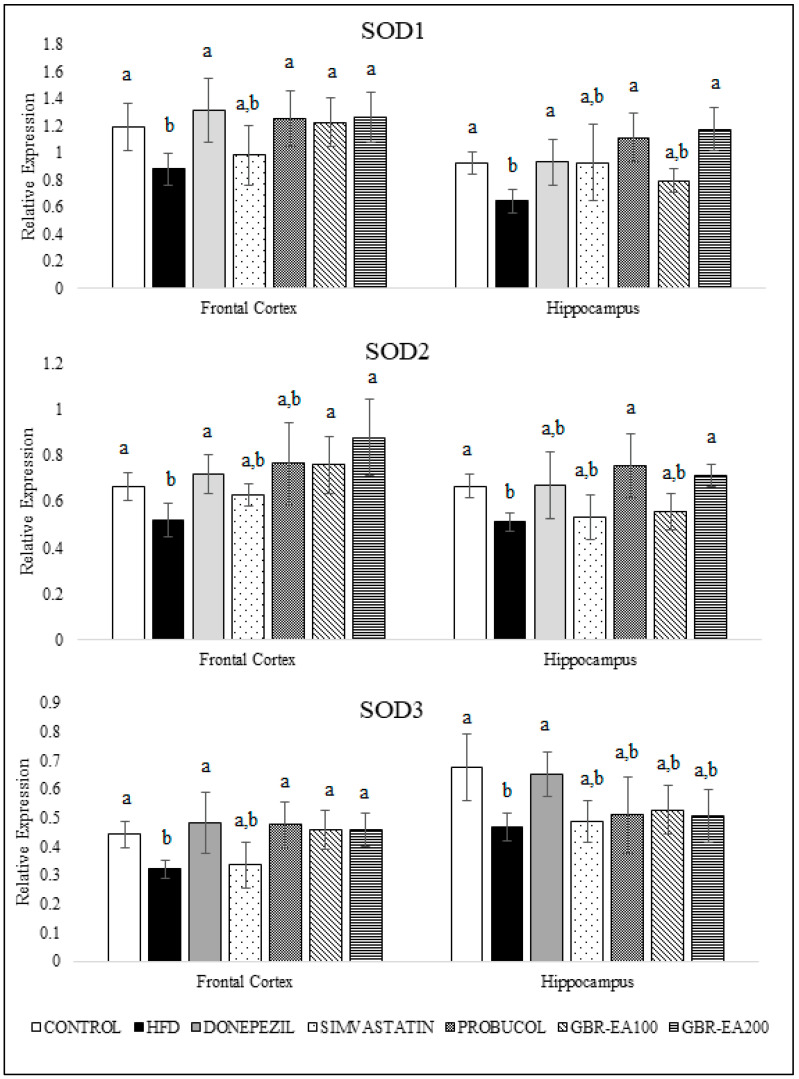
Expression level of superoxide dismutases (SODs) in the frontal cortex and hippocampus of rats fed a high-fat diet (HFD) for 6 months, as determined using a multiplex GeXP analysis system. Values represent the mean ± SD. a–b Mean values with different letters were significantly different among the groups (*p* < 0.05).

**Figure 4:**
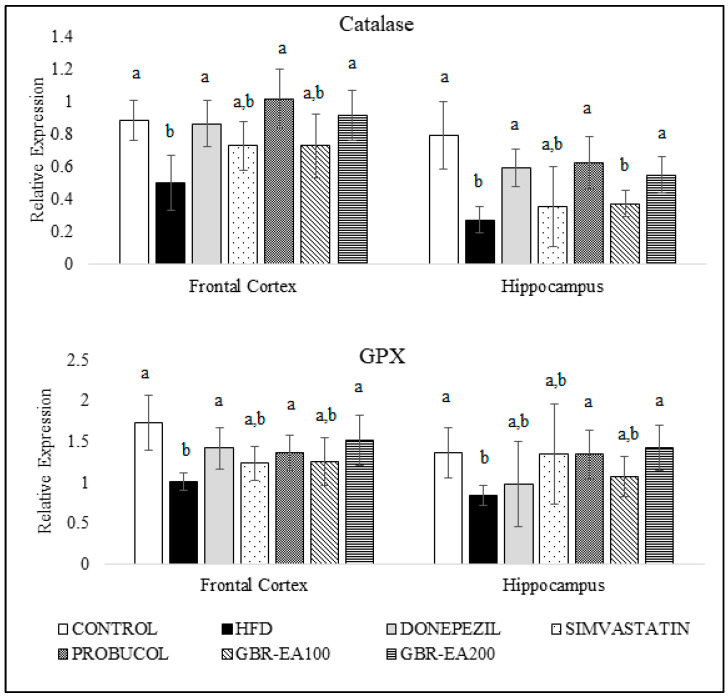
Expression level of catalase and GPX in the frontal cortex and hippocampus of rats fed a high-fat diet (HFD) for 6 months, as determined using a multiplex GeXP analysis system. Values represent the mean ± SD. a–b Mean values with different letters were significantly different among the groups (*p* < 0.05).

Figure 3 and Figure 4 show the expression of antioxidant genes, namely superoxide dismutases (SODs), catalase, and GPX, in both the frontal cortical and hippocampal regions of the rats’ brains. The current study showed that consumption of a high-cholesterol diet markedly suppressed SOD1, SOD2, and SOD3, as well as catalase and GPX in both the frontal cortex and hippocampus areas (*p* < 0.05). In contrast, treatment with GBR-EA (100 mg/kg and 200 mg/kg) significantly elevated the expression of all antioxidant genes, namely SOD1, SOD2, SOD3, catalase, and GPX in the frontal cortex, and SOD1, SOD2, catalase, and GPX in hippocampus, when compared to the HFD group (*p* < 0.05). However, GBR-EA at both doses did not show any significant difference compared to the HFD group for SOD3 expression in the hippocampus (*p* > 0.05). It was noted that the increment caused by GBR-EA was in a dose-dependent manner in the hippocampus for SOD1, while similar effects were observed for SOD2, catalase, and GPX in both the cortex and hippocampus. In the hippocampus, there was no significance difference for the expression of SOD2 and GPX among the GBR-EA100 and GBR-EA200 groups (*p* > 0.05). All GBR-EA groups showed no significant difference in terms of their frontal cortical catalase and hippocampal SOD3 expression (*p* > 0.05). Interestingly, treatment with Simvastatin did not show any significant differences when compared with HFD, in terms of all antioxidant genes (SOD1, SOD2, SOD3, catalase, and GPX), in both regions (*p* > 0.05).

It is recognized that oxidative stress is a major factor associated with the development and progression of AD and other forms of dementia. The brain is enriched with readily peroxidizable polyunsaturated fatty acids, and is preferentially susceptible to oxidative stress [49] due to its high oxygen consumption. Moreover, unlike other organs, the brain is not particularly well equipped with high antioxidant defenses. It has a low level of catalase and only moderate amounts of the endogenous antioxidant enzymes SOD and GPX. The hippocampus is the site of structural abnormalities associated with the early stages of AD and other cognitive dementias. The hippocampus, which is responsible for memory and cognition, is highly susceptible compared to other brain regions to a variety of insults including environmental toxicants, vascular risk factors, and metabolic perturbations [26,50], and was found to be susceptible to the oxidative stress caused by hypercholesterolemia [26,51].

The results from the current investigation, as well as those from other published studies, demonstrate that the consumption of a high-fat/cholesterol diet not only affects the peripheral vasculature, as implied by the serum antioxidant and biochemical profile, but also induces oxidative changes in these brain regions [26,34,51]. In this study, HFD consumption reduced SODs, catalase, and GPX in both the frontal cortical and hippocampal regions of the brain, implying a reduced defense against oxidative stress in both areas, which are highly prone to being affected in AD cases. In addition, it was revealed that SOD1 downregulation and deficiency promoted Aβ oligomerization and memory loss in a mouse model of AD [52,53]. Oxidative stress in the brain caused by excessive cholesterol intake was evidenced by the suppressed expression of antioxidant genes, as a result of overwhelming toxic stimuli that are able to surpass the cellular defense system. Therefore, the upregulation of antioxidant defenses is an attempt to boost endogenous antioxidants, to prevent oxidative damage on cells [54].

Interventions with antioxidants such as GBR exhibited neuroprotection, by which they are able to boost the cellular mechanism for clearing the free radicals and counteract the oxidative stress. The neuroprotective properties of GBR have been partly reported, and the mechanisms underlying its effects on the brain remain to be determined. The results of this current study indicate that the effect of GBR against oxidative stress may be mediated by the induction of endogenous antioxidant defenses through the upregulation of SOD1, SOD2, SOD3, catalase, and GPX, in both the frontal cortex and hippocampus. From this study, the upregulation of SODs by GBR and GBR-EA implies that both treatments efficiently regulate the conversion of O_2_^−^ to the less reactive H_2_O_2_. The cumulative effects of GBR-EA are most likely due to the higher levels of bioactive contents in the extract, such as phenolics and oryzanol constituents, which have already been reported to possess high antioxidant properties [25,39].

**Figure 5:**
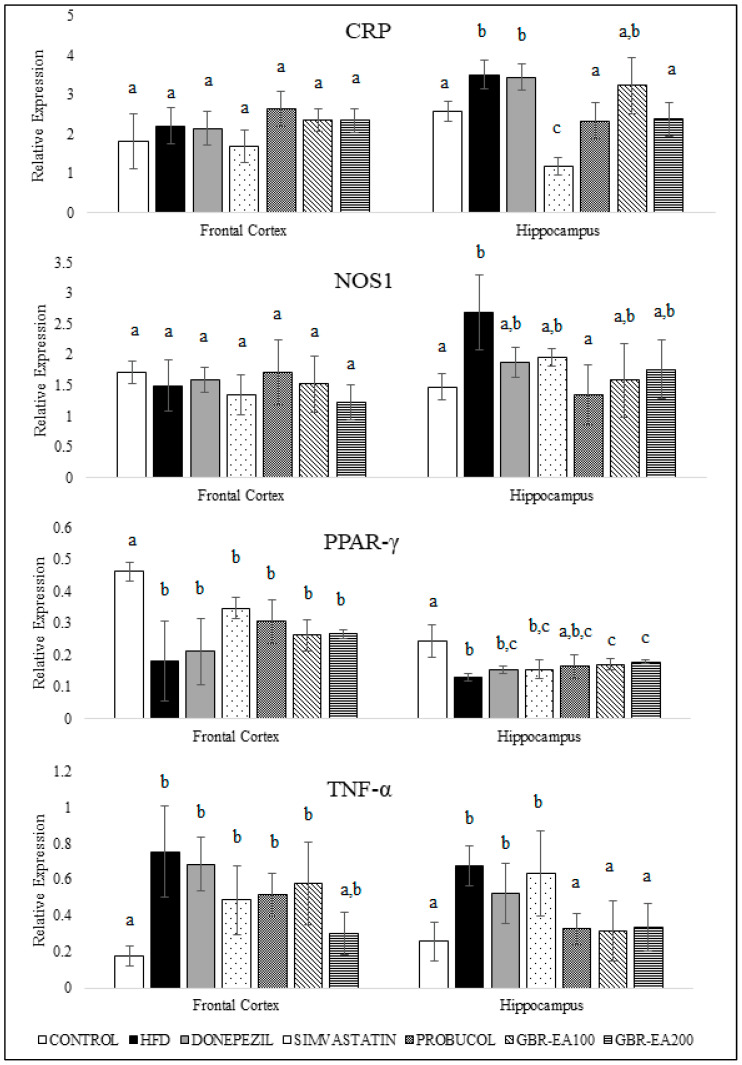
Expression level of CRP, NOS1, PPAR-γ, and TNF-α in the frontal cortex and hippocampus of rats fed a high-fat diet (HFD) for 6 months, as determined using a multiplex GeXP analysis system. Values represent the mean ± SD. a–c Mean values with different letters were significantly different among the groups (*p* < 0.05).

Figure 5 shows the expression of genes involved in the inflammatory pathway, namely CRP, NOS1, PPAR-γ, and TNFα. It was found that the expression of CRP and NOS1 in the frontal cortex showed no changes between groups (*p* > 0.05), while the expression of both genes was significantly elevated in the hippocampal region for the HFD group as compared to the control (*p* < 0.05). Treatment with Simvastatin and Probucol significantly attenuated the HFD-induced CRP elevation in the hippocampal area (*p* < 0.05), while the Donepezil group exhibited a similar expression to the HFD group. Treatment with GBR-EA200 showed a significant decrease in the expression of CRP (*p* < 0.05), when compared to HFD. Treatment with Probucol significantly lowered the expression of NOS1 compared with HFD (*p* < 0.05). However, no significant difference was noted in the expression of NOS1 for Donepezil and Simvastatin compared to HFD. The consumption of HFD was found to reduce the expression of PPAR-γ in both the frontal cortex and hippocampus, when compared to the normal control (*p* < 0.05). However, in the frontal cortex, no significant differences were found for all intervention groups compared to the HFD group (*p* > 0.05). On the other hand, in the hippocampal area, the expression of PPAR-γ was significantly elevated in the groups treated with GBR-EA at 100 and 200 mg/kg BW, when compared with HFD (*p* < 0.05). However, the expression was not significantly different when comparing among all the treated groups (*p* > 0.05). Meanwhile, no significant difference was found for the treatments with Donepezil, Simvastatin, and Probucol when compared with HFD (*p* > 0.05).

On the other hand, the HFD group showed a significant increase in the expression of TNF-α for both the frontal cortex and hippocampus compared to the control (*p* < 0.05). No changes were observed for Donepezil and Simvastatin in the frontal cortical and hippocampal region. Treatment with Probucol tended towards lowering the TNF-α expression. Even though the expression was not significantly different from the HFD group in the frontal cortex (*p* > 0.05), Probucol was found to reduce the expression of TNF-α significantly in the hippocampus (*p* < 0.05). In addition, this study revealed that the TNF-α expression was decreased significantly in both regions for groups treated with GBR-EA100 and GBR-EA200, when compared to HFD (*p* < 0.05), thus implying their anti-inflammatory actions. It has already been suggested that PPAR-γ and its agonists will increase SOD, catalase, and IDE expressions, which are protective to the brain [51,55].

Interestingly, the transcriptional changes of inflammatory biomarker expressions in the frontal cortex and hippocampus of the HFD group in this study were consistent with an increased risk of neurodegenerative diseases. The findings from this study showed that hypercholesterolemia resulted in inflammatory processes in the frontal cortex and hippocampus, in which the induction with cholesterol reduced the expression of PPAR-γ in both regions. Both experimental and clinical studies have shown that brain function is sensitive to inflammatory mediators, which may involve various mechanisms, including an acute phase response to damaged tissue, as well as a response to accumulated Aβ [26,56]. High levels of serum cholesterol not only induce vascular changes similar to the early inflammatory lesions of atherosclerosis but, in addition, induced the blood–brain barrier permeability and localized neuroinflammatory changes observed [57]. Brain microvessels isolated from AD patients presented high levels of cytokines and chemokines, thus supporting the contribution of cerebrovascular inflammation in AD pathogenesis [58]. In addition, the highest levels of cytokine binding were demonstrated in certain areas associated with learning and memory, including regions of the cortex and hippocampus [59,60].

In the current study, Donepezil exhibited potential antioxidant properties in an HFD-induced animal model. It was found that Donepezil’s antioxidant regulation involves the regulation of SODs, catalase, and GPX. The antioxidative effects of Donepezil were previously documented [52,61], thus supporting the findings from the present study. Indeed, Donepezil is known to inhibit AChE enzymes, thus causing an increased level of ACh in the synaptic cleft. ACh has been reported to regulate NO levels in the brain and confer neuroprotective properties [62,63]. However, Donepezil did not alter the regulation of inflammatory-related biomarkers such as CRP, TNFα, NOS1, and PPARγ.

Previous findings showed that increased biomarkers such as CRP, NOS1, and TNF-α in the brain resulted in a high susceptibility to neurodegeneration [64,65,66]. Consistent with this notion, and based on the relevance of CRP and Aβ in transgenic mice and in vitro models, it was demonstrated that CRP is a potential trigger of Aβ production, thus it is speculated that CRP may be elevated with Aβ pathogenesis in the early stages of AD [67,68]. CRP inhibitor effectively inhibited the CRP-induced upregulations of Aβ-related production, thus providing important evidence of their associations; therefore, CRP may be a novel target for early AD intervention [67,68]. Furthermore, not only TNF-α inhibits the transport of Aβ from the brain to the periphery, elevated TNF-α levels may increase the brain accumulation of Aβ [69,70]. Moreover, the secretion of Aβ may also be further enhanced by TNF-α, thus resulting in a vicious cycle between Aβ and TNF-α in the AD brain [71]. As exhibited by previous findings, this present study supported the antioxidant effects of Probucol in the brain. In this animal model, Probucol was found to upregulate the expression of all antioxidant genes, namely SODs, catalase, and GPX. The neuroprotective effects of Probucol in the brain might also be explained by its anti-inflammatory effects through the regulation of NOS1, CRP, and TNFα, particularly in the hippocampus.

### 3.5. Effects of GBR-EA on Hippocampal Acetylcholinesterase Gene and Enzyme Level

**Figure 6:**
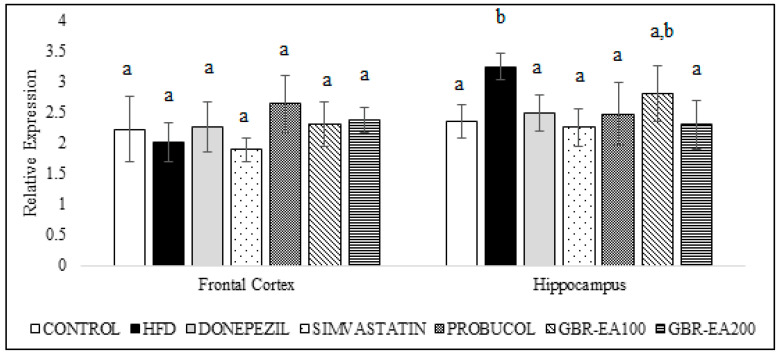
Expression level of acetylcholinesterase in the frontal cortex and hippocampus of rats fed a high-fat diet (HFD) for 6 months, as determined using a multiplex GeXP analysis system. Values represent the mean ± SD. a–b Mean values with different letters were significantly different among the groups (*p* < 0.05).

**Figure 7:**
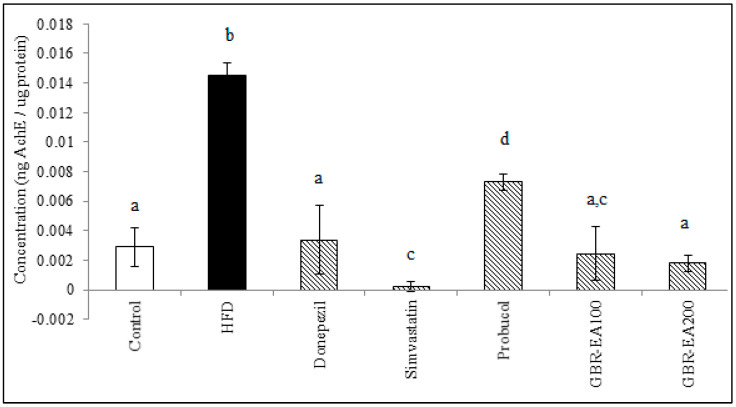
Acetylcholinesterase level in the hippocampus of rats fed with a high-fat diet (HFD) for 6 months, as determined by ELISA. Values represent the mean ± SD. a–d Mean values with different letters were significantly different among the groups (*p* < 0.05).

As shown in Figure 6, the expression of AChE mRNA did not differ among all groups in the frontal cortex. However, in the hippocampus, the expression was significantly increased with the consumption of HFD compared to the control (*p* < 0.05). With treatment with AChE inhibitor, Donepezil was found to downregulate the expression of AChE compared to HFD (*p* < 0.05). Simvastatin, Probucol, and GBR-EA (particularly at 200 mg/kg) also showed significantly reduced expressions of the gene (*p* < 0.05). Furthermore, the level of AChE in the hippocampus was measured, and it was found that the protein level was significantly increased in HFD group when compared to the normal control group (Figure 7). In concordant with the mRNA expressions, treatment with Donepezil, an AChE inhibitor, as well as Simvastatin and GBR-EA at both doses, markedly reduced AChE levels (*p* < 0.05). However, even though the mRNA expression was elevated for Probucol, the groups showed a significantly reduced level of AChE enzyme compared to the HFD group (*p* < 0.05). There was no significance difference between the GBR-EA groups, when compared among the two groups (*p* > 0.05).

Elevated AChE is proposed as an early event associated with hypercholesterolemia-induced cognitive impairments [16,72]. In a previous study, the AChE level in the hippocampus suggested that a high level of fat in the diet for a period of 12 weeks did not disrupt the cholinergic system [73]. It should be noted that lowered hippocampal AChE levels have been observed after long-term consumption of a high-fat diet [74], whilst a cholesterol-enriched diet showed decreased ChAT-positive neurons in the basal nucleus [15], thereby suggesting longer term manipulation may be necessary. Thus, in this present study, HFD was induced for 3 months before starting the intervention, together with HFD for another 3 months. A possible interaction was suggested between cholesterol and membrane-bound cholinergic receptors, and thus increased cholesterol levels may modulate their cholinergic function.

HFD consumption was reported to increase AChE enzyme activity in the brain, and this study showed similar results in the expression of the AChE gene and the increased AChE level in hippocampus, as measured by ELISA. The anticholinesterase effect of Simvastatin in the present study also corroborated that of previous findings [75,76]. A similar result was obtained in that study for the use of Donepezil, a cholinesterase inhibitor, thus validating the current findings. GBR-EA extract has shown promising effects on the inhibition of AChE gene expression, as well as in its activity in the rat hippocampus, suggested to be due to the presence of certain compounds, particularly ferulic acid and oryzanol components [24], that have been reported to inhibit AChE competitively [77,78] and are likely to possess a binding ability with the AChE structure.

Previous studies have shown that hypercholesterolemia is commonly accompanied by antioxidant deregulation, oxidative damage, inflammation, and altered cholinergic signaling in the brain. These led to cognitive alteration, due to the impairment of areas associated with learning and memory processes, particularly the prefrontal cortex and hippocampus [16,33,51]. The present study demonstrated that the neuroprotective effects of GBR may partly be regulated through modifications of antioxidants (SODs, catalase, and GPX) and attenuation of the inflammatory process in the hippocampus. Nevertheless, the underlying mechanism of AD pathogenesis remains elusive, and other than neuroinflammation, AD patients are also characterized by amyloid plaques, neurofibrillary tangles of tau protein, loss of neuronal connections, and cell death, as well as mitochondrial dysfunction and proteasome inhibition [79,80]. While the accumulated knowledge demonstrates that modulation of the cholinergic system might be beneficial for attenuating the development of Aβ formation, and restoring cholinergic neurotransmission and consequently improving cognition in AD, future studies could be directed at investigating the effect of GBR and its extracts in modulating other AD pathogeneses.

## 4. Conclusions

In conclusion, the results from this study reinforce the link between diet-induced hypercholesterolemia with molecular perturbation in the brain, highlighting the role of oxidative stress and inflammation. More importantly, this study also demonstrated that GBR-EA is neuroprotective against cholesterol-induced damage in the brain, as evidenced by the improved biochemical profile, increased serum antioxidant, and the regulatory effects on antioxidant (SODs, catalase, and GPX) gene expression. The neuroinflammatory alterations in the hippocampus were improved by GBR-EA through a reduction of AChE levels, as well as the downregulation of CRP and TNF- α and upregulation of PPAR-γ mRNA expressions. The findings from the current study also substantiated the use of adult hypercholesterolemic rats as a model to study neurodegenerative changes in the brain, especially at the molecular level.

## Figures and Tables

**Table 1:** Diet composition and treatments.

Group	Oral Gavage Treatment	Food Composition					Calorie (kcal/100 g Food)
		Normal Pellet	Oil	Corn Starch	Cholesterol	Cholic Acid	
Control	Normal saline	100%	-	-	-	-	335
HFD	Normal saline	65%	20%	10%	4.5%	0.5%	437.75
Donepezil	1.5 mg/kg BW Donepezil	65%	20%	10%	4.5%	0.5%	437.75
Simvastatin	10 mg/kg BW Simvastatin	65%	20%	10%	4.5%	0.5%	437.75
Probucol	200 mg/kg BW Probucol	65%	20%	10%	4.5%	0.5%	437.75
GBR-EA100	100 mg/kg BW GBR-EA extract	65%	20%	10%	4.5%	0.5%	437.75
GBR-EA200	200 mg/kg BW GBR-EA extract	65%	20%	10%	4.5%	0.5%	437.75

**Table 2:** Gene, accession number, and reverse and forward primer sequences used in GeXP Multiplex Gene Expression Analysis.

Gene	Accession Number	Primer Sequences with Universal Tags (Underlined)	
		Forward (5′-3′)	Reverse (3′-5′)
ACTB ^a^	NM_031144	AGGTGACACTATAGAATAGGCATCCTGACCCTGAAGTA	GTACGACTCACTATAGGGAAGACGCAGGATGGCATGAG
Atp50a	NM_138883	AGGTGACACTATAGAATACTCTCTGAGTTAAAGACAGTGCTGA	GTACGACTCACTATAGGGAACAATCATCCCACCCATGAT
Cyclophilin A ^a^	NM_017101	AGGTGACACTATAGAATATTCTGTAGCTCAGGAGAGCA	GTACGACTCACTATAGGGATTGAAGGGGAATGAGGAAAA
GAPDH ^a,#^	NM_017008	AGGTGACACTATAGAATACTGAGGACCAGGTTGTCTCC	GTACGACTCACTATAGGGAGAGGGCCTCTCTCTTGCTCT
Kan(r) ^b^	-		
AChE	NM_172009	AGGTGACACTATAGAATAAGTTCGACCACTATAGCAAG	GTACGACTCACTATAGGGAAAGATGAGGATCCCCTAGT
Catalase	NM_012520	AGGTGACACTATAGAATAACTGCAAGTTCCATTACAAG	GTACGACTCACTATAGGGAGTTCAACTTCAGCAAAATAAT
CRP	NM_017096	AGGTGACACTATAGAATACTAAACAGGCCTTCGTATT	GTACGACTCACTATAGGGACAAGCCAAAGCTCTACAAT
GPX	NM_030826	AGGTGACACTATAGAATATTGAGAAGTTCCTGGTAGGT	GTACGACTCACTATAGGGATTTTCTGGAAATCAGGTGT
NOS1	NM_052799	AGGTGACACTATAGAATAAACTCTCGATACAACATCCT	GTACGACTCACTATAGGGACTTGTCACTCTGGAAGCTA
PPAR-γ	NM_013124	AGGTGACACTATAGAATAAAATCTCTGTTTTATGCTGTTA	GTACGACTCACTATAGGGACAACCATGGTAATTTCTTGT
SOD1	NM_017050	AGGTGACACTATAGAATATCAATATGGGGACAATACAC	GTACGACTCACTATAGGGATACTTTCTTCATTTCCACCTT
SOD2	NM_017051	AGGTGACACTATAGAATATGTATGAAAGTGCTCAAGAT	GTACGACTCACTATAGGGAGCCCTCTTGTGAGTATAAGT
SOD3	NM_012880	AGGTGACACTATAGAATATCGAACTACTTTATGCCC	GTACGACTCACTATAGGGAGAAGACAAACGAGGTCTCTA
TNF-α	NM_012675	AGGTGACACTATAGAATACCCAACAAGGAGGAGA	GTACGACTCACTATAGGGATGGTGGTTTGCTACGA

Based on the *Rattus norvegicus* gene sequences adopted from the National Center for Biotechnology Information GenBank Database. ^a^ Housekeeping genes. ^b^ Internal control supplied by Beckman Coulter Inc (Miami, FL, USA) as part of the GeXP kit. ^#^ Normalization gene. Underlined sequences are forward and reverse universal sequences (tags). ACTB: beta-actin; AChE: Acetylcholinesterase; GAPDH: Glyceraldehyde-3-Phosphate Dehydrogenase; CRP: C-reactive protein; GPX: glutathione peroxidase; NOS1: Nitric Oxide Synthase 1; Kan(r): Kanamycin resistant; PPAR: Peroxisome proliferator-activated receptor; TNF: tumor necrosis factor; SOD: superoxide dismutase.

**Table 3:** Food intake and body weight gain of experimental rats.

Groupings	Food Intake	Initial Weight (g)	Final Weight (g)	Weight Gain (g)
	(kcal/100 g BW/day)			
Normal	15.57 ±6.48 ^a^	259.63 ± 12.33 ^a^	424.25 ± 19.09 ^a^	164.63 ± 11.92 ^a^
HFD	16.58 ± 6.44 ^a^	261.00 ± 29.03 ^a^	479.80 ± 43.72 ^a^	218.80 ± 27.34 ^b^
Donepezil	18.73 ± 6.81 ^a^	262.50 ± 14.39 ^a^	422.50 ± 22.12 ^a^	160.00 ± 28.16 ^a,b^
Simvastatin	18.78 ± 2.68 ^a^	257.25 ± 19.92 ^a^	425.00 ± 31.94 ^a^	167.75 ± 38.14 ^a,b^
Probucol	17.12 ± 6.17 ^a^	274.71 ± 14.26 ^a^	448.71 ± 28.76 ^a^	174.00 ± 24.41 ^a,b^
GBR-EA100	17.37 ± 6.33 ^a^	252.18 ± 21.55 ^a^	426.00 ± 39.06 ^a^	173.82 ± 34.11 ^a,b^
GBR-EA200	18.65 ± 7.60 ^a^	260.00 ± 22.01 ^a^	426.33 ± 38.52 ^a^	166.33 ± 27.45 ^a,b^

Data are represented as mean ± SD. ^a–b^ letters in columns indicate a statistically significant difference among different groups (*p* < 0.05).

## Data Availability

The data that support the findings of this study are available from the corresponding author upon reasonable request.

## Sample Availability

Samples of the compounds are not available from the authors.
